# Determination of Chiral Impurity of Naproxen in Different Pharmaceutical Formulations Using Polysaccharide-Based Stationary Phases in Reversed-Phased Mode

**DOI:** 10.3390/molecules27092986

**Published:** 2022-05-06

**Authors:** Lajos-Attila Papp, Sarolta Krizbai, Máté Dobó, Gabriel Hancu, Zoltán-István Szabó, Gergő Tóth

**Affiliations:** 1 Department of Pharmaceutical and Therapeutic Chemistry, Faculty of Pharmacy, George Emil Palade University of Medicine, Pharmacy, Science, and Technology of Targu Mures, 540142 Targu Mures, Romania; lajos.papp@umfst.ro (L.-A.P.); krizbai.sarolta@gmail.com (S.K.); gabriel.hancu@umfst.ro (G.H.); 2 Department of Pharmaceutical Chemistry, Semmelweis University, 1092 Budapest, Hungary; dobomate99@gmail.com (M.D.); toth.gergo@pharma.semmelweis-univ.hu (G.T.); 3 Department of Pharmaceutical Industry and Management, Faculty of Pharmacy, George Emil Palade University of Medicine, Pharmacy, Science, and Technology of Targu Mures, 540142 Targu Mures, Romania

**Keywords:** naproxen, chiral separation, method optimization, polysaccharide stationary phase, reversed-phase HPLC, experimental design

## Abstract

A novel, validated, reversed-phase (RP), chiral high performance liquid chromatography (HPLC) method was developed for the enantiopurity control analysis of naproxen, a frequently used non-steroidal anti-inflammatory agent using polysaccharide-type chiral stationary phase (CSP). In the screening phase of method development, seven columns were tested in polar organic (PO) mode using mobile phases consisting of 0.1% acetic acid in methanol, ethanol, 2-propanol, and acetonitrile. Enantiorecognition was observed only in five cases. The best enantioseparation was observed on a Lux Amylose-1 column with 0.1% (*v*/*v*) acetic acid in ethanol with a resolution (*R*_s_) of 1.24. The enantiomer elution order was unfavorable, as the distomer eluted after the eutomer. When the ethanolic mobile phase was supplemented with water, enantiomer elution order reversal was observed, indicating a difference in the enantiorecognition mechanism upon switching from PO to RP mode. Furthermore, by changing ethanol to methanol, not only lower backpressure, but also higher resolution was obtained. Subsequent method optimization was performed using a face-centered central composite design (FCCD) to achieve higher chiral resolution in a shorter analysis time. Optimized parameters offering baseline separation were as follows: Lux Amylose-1 stationary phase, thermostated at 40 °C, and a mobile phase consisting of methanol:water:acetic acid 85:15:0.1 (*v*/*v*/*v*), delivered with 0.65 mL/min flow rate. Using these optimized parameters, a *R*_s_ = 3.21 ± 0.03 was achieved within seven minutes. The optimized method was validated according to the ICH guidelines and successfully applied for the analysis of different pharmaceutical preparations, such as film-coated tablets and gel, as well as fixed-dose combination tablets, containing both naproxen and esomeprazole.

## 1. Introduction

**Figure 1:**
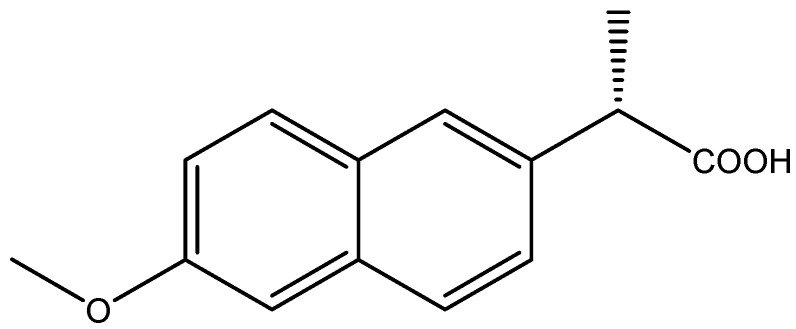
The chemical structure of Naproxen ((*S*)-(+)-6-Methoxy-α-methyl-2-naphthaleneacetic acid).

Naproxen, an aryl propionic acid (profen) nonsteroidal anti-inflammatory drug (NSAID), was first approved in 1976 and has been used in pain therapy for more than 45 years. It is used in therapy as an analgesic, anti-inflammatory, and antipyretic drug [1,2]. Just in the United States of America, it is estimated that the number of prescriptions was more than 11.5 M, while the number of patients was more than 4.5 M in 2019 [3]. The molecule contains one asymmetric carbon atom, and it exists in two enantiomeric forms (*R*-naproxen, *S*-naproxen). The structure of naproxen is depicted in Figure 1.

Naproxen is used as a single enantiomer, in the form of *S*-naproxen. *S*-naproxen exhibits higher affinity toward cyclooxygenase (COX) than its distomer, while *R*-naproxen is hepatotoxic. Although naproxen was discovered over 50 years ago, asymmetric approaches are still being developed for its synthesis, and these should be supported by modern chiral analytical separation techniques. Nevertheless, the official United States Pharmacopoeia (USP) monograph does not contain a chromatographic enantiopurity analytical method for this drug, as only the determination of the specific optical rotation value is stipulated. The European Pharmacopeia (EurPh) monograph uses a Pirkle-type stationary phase and normal phase high performance liquid chromatography (HPLC) method for the determination of the enantiomeric purity of naproxen. According to EurPh regulations, the *R*-naproxen content should not exceed 2.5%, which can be considered a relatively high limit value in comparison to the majority of enantiopure pharmaceuticals. Studies applying capillary electrophoresis (CE) using cyclodextrin (CD) [4,5,6,7] or vancomycin/chiral ionic liquid synergistic system [8] as chiral selectors (CSs), as well as HPLC using polysaccharide-based chiral stationary phases (CSPs) [9,10,11,12,13,14,15,16,17,18], have been published so far for the chiral separation of naproxen. Although CE has some advantages over HPLC, such as low organic solvent and sample consumption, its relatively low sensitivity and generally lower reproducibility hinders its widespread application in the routine analysis of small molecules. Direct HPLC using CSPs remains the golden standard for enantiopurity analysis. Although the development of CSPs is ongoing and more and more products are available on the market, polysaccharide phenylcarbamates and esters-based CSPs introduced by Okamoto et al. in the 1980s are the most frequently used CSs due to their high success rate and multimodal mechanism of separation. These columns can be used in normal-phase (NP), reversed-phase (RP), and polar organic (PO) modes [19,20].

Taking a closer look at the previously published HPLC methods for chiral analysis of naproxen, it can be observed that many of these use NP chromatography with toxic, polluting organic solvents [9,10,11,13,14]. Besides, only a few HPLC methods dedicated to the enantiomeric purity control of naproxen using polysaccharide CSPs are available. Tanaka et al. developed a RP HPLC method applicable for the simultaneous determination of the enantiomeric purity and several related achiral impurities of naproxen. The optimized method employed Chiralpak AS-3R column (containing amylose tris((*S*)-methylbenzylcarbamate)) CS coated on 3 µm silica gel) in combination with a mobile phase consisting of a mixture of phosphate buffer and acetonitrile (ACN) [12]. Ragab et al. reported a NP HPLC method using Kromasil Cellucoat (containing cellulose tris(3,5-dimethylphenylcarbamate) CS) column for the simultaneous enantiomeric purity testing of *S*-naproxen and esomeprazole in combination tablets. The method was validated for the stereoselective determination of the *S*-isomers of each drug in the presence of their chiral impurity, and it proved to be capable of determining their distomers at a 1% relative concentration level [13]. A few studies dealing with the enantiomeric determination of naproxen from environmental samples using the liquid chromatography coupled with mass spectrometry (LC-MS) technique are also available [15,16].

This study aimed to develop a novel, rapid, and selective HPLC method for the enantiomeric purity control of naproxen, applicable in the routine analysis of different solid and semisolid pharmaceutical preparations, as well as of combination dosage forms, using polysaccharide-based CSPs. Polysaccharide-type CSPs are widely applied for chiral analysis in HPLC due to their versatility. Because of the several disadvantages of NP methods (specific instruments and columns and the employed solvents are generally toxic), we aimed to develop a method in PO and/or RP modes. Matarashvili et al. observed a reversal of enantiomer elution order for several acidic analytes based on water content in the mobile phase, which could be exploited in enantiomeric quality control, where the appropriate elution order (distomer first) is crucial [21]. This behavior could be useful for the enantiopurity analysis of naproxen, as we further aimed to validate the developed method and test its applicability on different dosage forms. A combination formulation was also tested, where the possibility of testing for the chiral impurity of both naproxen and esomeprazole was investigated.

## 2. Results

### 2.1. Screening Phase

**Figure 2:**
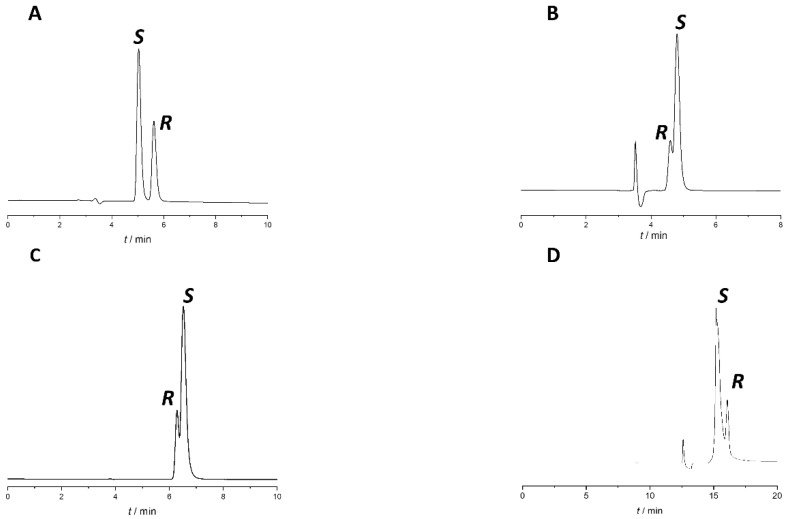
Representative chromatograms from the method screening phase. (**A**) Lux Amylose-1 with MeOH:acetic acid 100:0.1 (*v*/*v*) (**B**) Lux Amylose-1 with ACN: acetic acid 100:0.1 (*v*/*v*), (**C**) Lux Cellulose-1 with MeOH:acetic acid 100:0.1 (*v*/*v*), (**D**) Lux Cellulose-4 with IPA:acetic acid 100:0.1 (*v*/*v*). All columns had identical dimensions: 150 mm × 4.6 mm, 5 µm. Other chromatographic parameters: flow rate: 0.5 mL/min, column temperature: 25 °C, detection at 230 nm.

Based on our early experiences [22,23,24,25] and literature data [26,27,28,29], polysaccharide-type CSPs with PO mode is one of the most successful pairings in chiral analyses. Therefore, in the screening phase, separation of naproxen enantiomers was attempted on seven polysaccharide-type CSPs, including amylose-based Lux Amylose-1, Lux i-Amylose-1, and Lux Amylose-2 and cellulose-based Lux Cellulose-1, Lux Cellulose-2, Lux Cellulose-3, and Lux Cellulose-4 in PO mode using a mobile phase consisting of 0.1% (*v*/*v*%) acetic acid in methanol (MeOH), ethanol (EtOH), 2-propanol (IPA), or ACN with a 0.5 mL/min flow rate at 25 °C. The results are summarized in Table 1. Representative chromatograms where enantiorecognition was observed are presented in Figure 2.

Enantiorecognition was observed in five cases from the twenty-four initial experiments, but none of the applied conditions offered baseline separation (*R*_s_ > 1.5) of the naproxen enantiomers. The best result was obtained using a Lux Amylose-1 column with EtOH:acetic acid 100:0.1 (*v*/*v*) (*R*_s_ ≈ 1.2). Unfortunately, in this case, enantiomer elution order (EEO) was not appropriate, as the distomer eluted after the eutomer. It is interesting to note that on Lux Amylose-1 using alcohol-type mobile phases, only the EtOH-based mobile phase showed enantiorecognition ability, as no enantioseparation using MeOH and IPA was observed. These results also underline the impact of different alcohols on the enantiorecognition mechanism on polysaccharide CSPs. On this column using EtOH and ACN, EEO reversal was observed, which could be explained by the different hydrogen-bond capabilities of the two solvents and their impact on the solvation and three-dimensional conformation of the CS [25,30,31].

**Figure 3:**
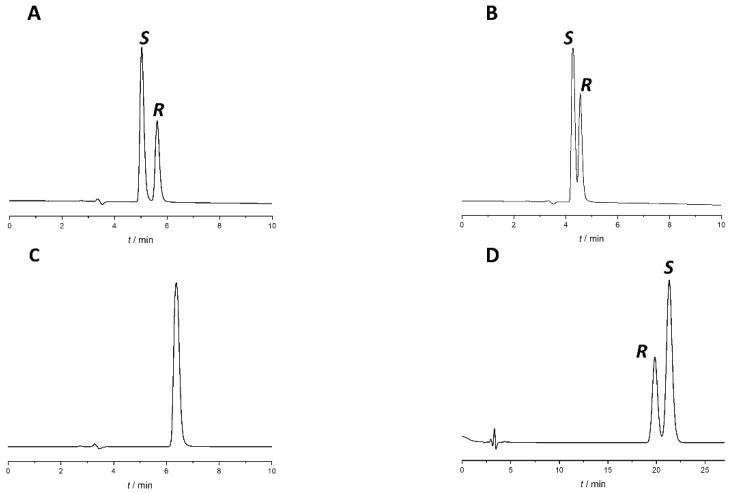
Effect of water content on separation of naproxen enantiomers using Lux Amylose-1 column. (**A**) EtOH:acetic acid 100:0.1 (*v*/*v*), (**B**) EtOH:water:acetic acid 90:10:0.1 (*v*/*v*/*v*), (**C**) EtOH:water:acetic acid 70:30:0.1 (*v*/*v*/*v*)), (**D**) EtOH:water:acetic acid 55:45:0.1 (*v*/*v*/*v*). Flow rate: 0.5 mL/min, column temperature: 25 °C, detection at 230 nm.

Changing the modifier type (acetic acid to formic acid) and concentration or changing other chromatographic parameters, such as temperature and flow rate, did not bring significant improvement to the separation. To increase *R*_s_, different percentages of acidified water were added to the most promising system, Lux Amylose-1 column with EtOH:acetic acid 100:0.1 (*v*/*v*) mobile phase. Upon the addition of the aqueous phase, there was a consequent transition from PO mode to RP mode. The chromatograms with different water percentages are presented in Figure 3.

**Figure 4:**
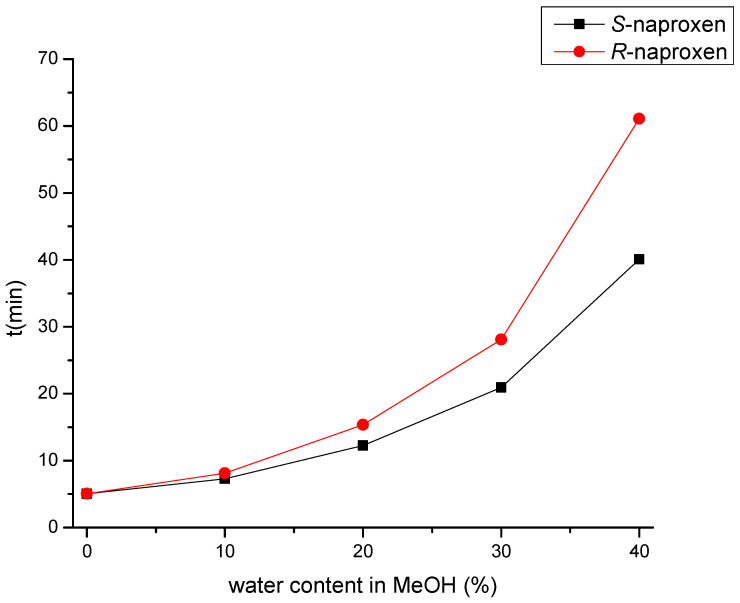
Retention profile of naproxen enantiomers in MeOH:water mixture on the Lux Amylose-1 column. All mobile phases contain uniformly 0.1% (*v*/*v*) acetic acid. Chromatographic conditions: flow rate: 0.5 mL/min, column temperature: 25 °C, detection at 230 nm.

As a result of water addition, the elution of the two enantiomers changed. This behavior allowed us to change the EEO and develop an appropriate method for *S*-naproxen purity control. A small amount of water reduces *R*_s_; when using a EtOH:water:acetic acid 70:30:0.1 (*v*/*v*) mobile phase, complete co-elution was observed, while even higher water content in the mobile phase reversed the EEO. This enabled the more favorable EEO, when the distomer *R*-naproxen eluted before the eutomer, *S*-naproxen. When using more than 30% water, the *R*_s_ increases with increasing water content. Applying a mobile phase composed of EtOH:water:acetic acid 55:45:0.1 (*v*/*v*) mixture baseline resolution with appropriate EEO was achieved. However, this mixture resulted in high column backpressure (~200 bar). By replacing EtOH to MeOH, even a smaller amount of water is enough to achieve baseline enantioseparation with adequate *R*_s_, appropriate EEO, and acceptable backpressure (less than 100 bar). Therefore, in the subsequent experiments, acidified MeOH:water mixture was used. Increasing the water proportion in the MeOH containing mobile phase was accompanied by both higher retention time and increased *R*_s_, resulting in a classical RP retention profile as seen in Figure 4.

Based on the screening phase results, the Lux Amylose-1 column with a mixture of MeOH:water:acetic acid 90:10:0.1 (*v*/*v*) was selected as a starting point for further method optimization using experimental design strategy.

### 2.2. Method Optimization

An experimental design (DoE) strategy was applied to optimize the analytical conditions and to investigate the effect of chromatographic parameters on enantioseparation. A face-centered central composite design (FCCD) using four analytical parameters with twenty-one experiments was employed in the design. The studied analytical parameters and their applied ranges were selected based on preliminary results: water content of the mobile phase (5–15% *v*/*v*) (factor A), column temperature (30–40 °C) (factor B), flow rate (0.5–0.7 mL/min) (factor C), and the percentage of acidic modifier in the mobile phase (0.05–0.15% *v*/*v*) (factor D). The experimental matrix and the obtained results are summarized in Table 2.

Resolution value between naproxen enantiomers (*R*_s_) and retention time of the second eluting enantiomer (*t*_2_) were selected as response variables.

A second-order polynomial model was applied, and analysis of variance (ANOVA) was carried out to estimate the significance of the model. The insignificant model terms were deleted one by one, re-evaluating the model after each deleted term. The following final regression models were obtained, in terms of coded factors, for the resolution and analysis time, respectively:

*R*_s_ = 2.59 + 1.03 * A − 0.080 * B − 0.24 * C − 0.037 * A * B − 0.14 * A * C + 0.037 * B * C (1)

*t*_2_ = 7.23 + 1.96 * A − 0.49 * B − 3.58 * C − 0.28 * A * B − 0.85 * A * C + 0.28 * B * C + 0.41 * A^2^
+ 1.31 * C^2^ (2)

The water content of the mobile phase had a great impact on both resolution and analysis time. By increasing the water content, higher resolution values and longer analysis times were observed, while higher flow rates decreased both resolution and analysis time. This factor had the most significant influence on the analysis time among the studied parameters, as expected, and a smaller impact on the resolution. The temperature had a smaller (even if statistically significant) contribution to the variations of both resolution and the analysis time in comparison to the water content of the eluent and the flow rate, respectively. Interestingly, in the case of the percentage of the acidic modifier in the mobile phase, no significant effect on the response variables was observed; thus, the final regression equations contain only three of the four studied chromatographic parameters.

As for performance indicators of the regression models, the values of *R*^2^ and *R*^2^*_adj_* were analyzed. For resolution, the *R*^2^ value was 0.9980 in accordance with the *R*^2^*_adj_* value of 0.9971. For analysis time, the *R*^2^ value of 0.9975 was also in good agreement with the *R*^2^*_adj_* value of 0.9958. In both cases, the *R*^2^ and *R*^2^*_adj_* values indicate that the models are suitable for navigation in the experimental domain.

**Figure 5:**
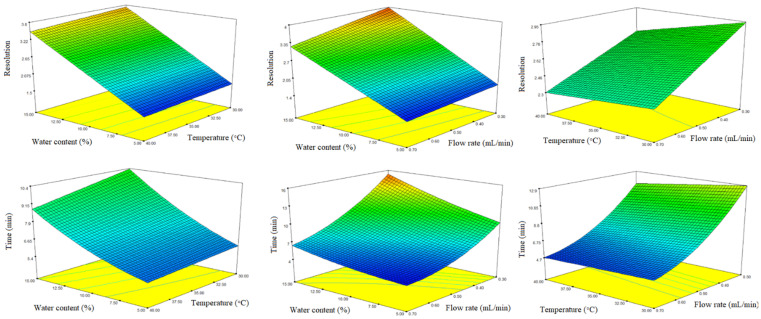
Three-dimensional response surface plots obtained for resolution and analysis time. Cold colors (blue) represent lower, while warm colors (red) represent higher values of the responses.

**Figure 6:**
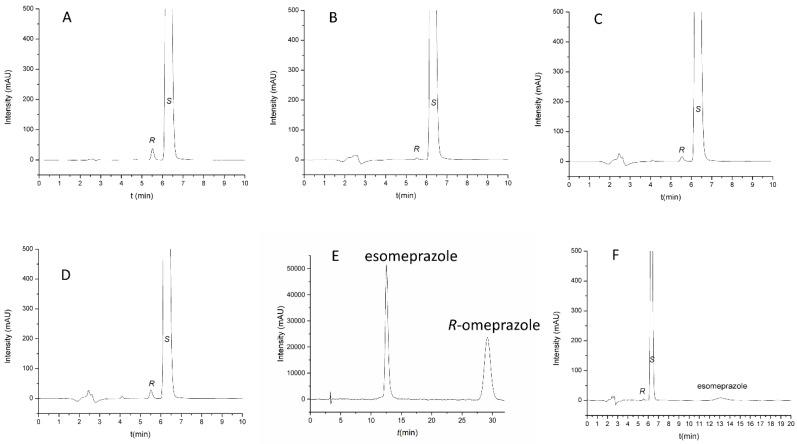
Representative chromatograms obtained during method application. (**A**) Naproxen sample spiked with 0.1% enantiomeric impurity; (**B**) Aleve, film-coated tablets; (**C**) Apranax, film-coated tablets; (**D**) Apranax Dolo gel; (**E**) Racemic omeprazole sample; (**F**) Vimovo delayed-release combination tablets. Chromatographic conditions: Lux Amylose-1 column, thermostated at 40 °C. Mobile phase: MeOH:water:acetic acid 85:15:0.1 (*v*/*v*/*v*). Flow rate 0.65 mL/min.

Three-dimensional response surface plots constructed based on the regression models are presented in Figure 5. To establish an optimal combination of the studied analytical conditions for both responses, the optimization feature of the statistical software was used, based on the application of the Derringer desirability function. Maximizing resolution and minimizing analysis time were set as objectives for method optimization. The factors were optimized with the same weight of priority. The optimal analytical conditions obtained, based on the desirability function, were as follows: Lux Amylose-1 stationary phase, thermostated at 40 °C, mobile phase consisting of MeOH/water/acetic acid 85:15:0.1 (*v*/*v*/*v*), and 0.65 mL/min flow rate. Using these optimal analytical conditions, the baseline resolution (*R*_s_ = 3.21 ± 0.03) of naproxen enatiomers was achieved within 7 min (Figure 6A).

### 2.3. Method Validation and Application

The method was validated according to guideline Q2(R1) of the International Council for Harmonization [32]. Linearity, sensitivity (expressed in limit of detection (LOD), and limit of quantification (LOQ)), accuracy and precision (expressed in reparability and intermediate precision) for the determination of *R*-naproxen as an enantiomeric impurity in the presence of the eutomeric *S*-antipode were investigated as the main important parameter in the validation. Method sensitivity was evaluated for the determination of *R*-naproxen, by sequentially diluting sample solutions. The LOD of *R*-naproxen was determined as the concentration yielding a signal 3:1 the baseline noise, while LOQ was determined at 10:1 signal to noise ratio in the presence of the eutomer. The LOQ was 2 μg/mL, while LOD for *R*-naproxen was 0.6 µg/mL. The reportable range of the developed method was set between 0.05 and 3% (LOQ to 120% of EurPh specification limit) regarding a target concentration of eutomer of 5 mg/mL. The validation data are summarized in Table 3. The linearity of the method was evaluated at nine concentration levels. Calibration plots were represented by plotting peak areas against corresponding concentrations (expressed in µg/mL). The correlation coefficient was determined by linear least-squares regression analysis. A 95% confidence interval of the y-intercepts included zero, and random distribution of the residuals was observed. Repeatability, intermediate precision, and accuracy were determined at three concentration levels. Results are summarized in Table 3. The obtained data proved that the optimized method is sensitive, linear, accurate, and precise for the determination of *R*-naproxen as enantiomeric impurity in *S*-naproxen samples.

The optimized and validated method was applied to the analysis of real pharmaceutical samples, in the form of film-coated tablets with a nominal content of 200 mg (as 220 mg naproxen sodium salt) and 250 mg (as 275 mg naproxen sodium salt), a gel containing 100 mg/g naproxen as well as fixed dose combination tablet containing 500 mg naproxen and 20 mg esomeprazole.

Representative chromatograms recorded for various samples are shown in Figure 6. Figure 6A displays the impurity-spiked sample solution of the active pharmaceutical ingredient. Figure 6B,C shows the representative chromatograms for the tested tablets, while the chromatogram displayed in Figure 6D is representative for the gel formulation. Figure 6E shows that our applied system is suitable for the separation of omeprazole enantiomers as well, proving the good selectivity and the ability of the method to be applied in the quality control of both esomeprazole and naproxen in one run. Figure 6F shows the representative chromatograms of combination tablets. It can be seen that all of the investigated products contain distomer impurity; however, its level is under 0.1% regardless of the type of the formulation, which is according to EurPh regulations. This result indicates the possibility to revise the actual limit of *R*-naproxen, as an impurity, in the EurPh monograph (2.5%), since our method allows for its determination at a much lower level. Thus, the presented method can represent a viable alternative to the compendial one due to its better reliability and lower LOQ value.

## 3. Materials and Methods

### 3.1. Materials

Naproxen and *R*-naproxen pharmaceutical impurity standard, as well as racemic omeprazole and esomeprazole magnesium hydrate, were purchased from Sigma-Aldrich, Hungary (Budapest, Hungary). HPLC-grade MeOH, EtOH, ACN, IPA, acetic acid, and formic acid were purchased from Merck (Darmstadt, Germany). The deionized water was prepared by a Milli-Q Direct 8 Millipore system. Lux Cellulose-1 (150 × 4.6 mm; particle size: 5 µm) [based on cellulose tris(3,5-dimethylphenylcarbamate)], Lux Cellulose-2 (150 × 4.6 mm; particle size: 5 µm) [based on cellulose tris(3-chloro-4-methylphenylcarbamate)], Lux Cellulose-3 (150 × 4.6 mm; particle size: 5 µm) [based on cellulose tris(4-methylbenzoate)], Lux Cellulose-4 (150 × 4.6 mm; particle size: 5 µm) [based on cellulose tris(4-chloro-3-methylphenylcarbamate)], Lux Amylose-1 (150 × 4.6 mm; particle size: 5 µm) [based on amylose tris(3,5-dimethylphenylcarbamate)], Lux i-Amylose-1 (150 × 4.6 mm; particle size: 5 µm) [based on amylose tris(3,5-dimethylphenylcarbamate)], and Lux Amylose-2 (Am2) (150 × 4.6 mm; particle size: 5 µm) [based on amylose tris(5-chloro-2-methylphenylcarbamate) were all the products of Phenomenex (Torrance, CA, USA).

The following pharmaceutical preparations: Apranax 275 mg (Valeant), Aleve (Bayer), Apranax dolo gel (Emo-Farm), were obtained from Central Pharmacy of Semmelweis University (Budapest, Hungary). Vimovo 500 mg/20 mg (Astra Zeneca) modified-release tablets were bought in a local pharmacy in Targu Mures, Romania.

### 3.2. LC-UV Analysis

Chromatographic experiments were performed on a Jasco HPLC system consisting of PU-2089 plus quaternary pump, AS-4050 autosampler, MD-2010 diode array detector, Jetstream 2 Plus thermostat. JASCO ChromNAV software was used for instrument control and data analysis. All separations were performed at 25 °C using 0.5 mL/min flow rate. UV detection was performed at 230 nm. In the screening phase, alcohols (MeOH, EtOH, or IPA) and ACN were used. Whenever an experiment required pre-treatment with either IPA, MeOH, EtOH, can, or alcohol–water mixture, it was brought about by pumping 10 column volumes (CV) of the corresponding solvent through the column.

All stock solutions were prepared at 1 mg/mL in MeOH, and further dilutions were performed with the same solvent. An injection volume of 5 µL was used, and three parallel measurements were carried out in each case. In preliminary experiments, *S*-naproxen spiked sample solutions were used. The final test solution of naproxen used for validation and method applicability testing was 5000 µg/mL. The impurity level percentages were calculated relative to this concentration.

For preparation of sample solutions, ten tablets were weighted, then ground, and mixed in a mortar. In a 25 mL volumetric flask, MeOH was added to an accurately weighted portion of the tablet powder corresponding to about 125 mg naproxen. In the case of gel formulation, a similar process was applied. Accurately weighed portion of pharmaceutical gel corresponding to 125 mg of naproxen was transferred into a 25 mL volumetric flask. The mixtures were placed into the ultrasonic bath for 30 min and then centrifuged for 10 min, applying 4000 rpm (Sartorius 2–16 P benchtop centrifuge, Goettingen, Germany). The clear supernatant was filtered through 0.22 μm pore size syringe containing PVDF filter (FilterBio membrane Co., LTD, Nantong City, China).

In the process of method optimization, Design Expert 7.0 statistical software (Stat-Ease, Minneapolis, MN, USA) was used for constructing the experimental plans and data evaluation.

## 4. Conclusions

A fast, chiral LC method was developed, optimized, and validated for the determination of enantiomeric purity of *S*-naproxen. Method development started in PO mode, exploring the effects of different solvents (MeOH, EtOH, IPA, ACN) on the chiral separation of naproxen enantiomers on seven different polysaccharide-based columns. In the applied chromatographic conditions, only weak enantiodiscrimination capabilities were observed, and none of the twenty-eight initially applied conditions resulted in baseline separation. In order to enhance *R*_s_, water was added to the mobile phase, which changed the elution mode from PO to RP mode. Increasing water content in the mobile phase not only enhanced retention time and *R*_s_, but it also resulted in EEO reversal, which led to the favorable distomer-first elution sequence. For subsequent fine tuning of the method, multivariate optimization through experimental design was undertaken, by applying a FCCD. The optimized method was subsequently validated and applied for the enantiomeric purity control of *S*-naproxen in a wide variety of pharmaceutical dosage forms, including film-coated tablets, gels, and combination tablets. The method proved to be selective, linear, accurate, and precise for the determination of *R*-naproxen as chiral impurity in naproxen-containing pharmaceuticals. Polysaccharide-based CSPs proved once again their true multimodal nature and their advantage in modulating enantioselectivity, through not only changing the CS, but also using different solvents and solvent combinations on the same CSP. Table 4 presents a comparison between analytical characteristics and results of our newly developed method and other previously published chiral separation methods in the literature for the enantioseparation of naproxen. Our work could be a guidance for the enantioseparation of other enantiopure NSAIDs from the profen (aryl propionic acids) class.

## Figures and Tables

**Table 1:** Chromatographic data from the screening phase—resolution (*R*_s_), enantiomer elution order (EEO), and retention time of second-eluting enantiomer (t_r2_).

CSP	Mobile Phase *	*t_r_*_2_ (min)	*R_s_*	EEO
Lux Amylose-1	MeOH	5.16	-	-
	EtOH	5.25	1.24	S > R
	IPA	5.68	-	-
	ACN	6.02	0.52	R < S
Lux i-Amylose-1	MeOH	4.42	-	-
	EtOH	4.46	-	-
	IPA	5.00	-	-
	ACN	4.52	-	-
Lux Amylose-2	MeOH	5.51	-	-
	EtOH	5.10	-	-
	IPA	6.43	-	-
	ACN	7.20	-	-
Lux Cellulose-1	MeOH	6.52	0.41	R > S
	EtOH	5.36	-	-
	IPA	7.05	-	-
	ACN	7.00	-	-
Lux Cellulose-2	MeOH	5.17	-	-
	EtOH	5.23	-	-
	IPA	7.74	-	-
	ACN	9.30	-	-
Lux Cellulose-3	MeOH	7.85	-	-
	EtOH	7.17	-	-
	IPA	9.20	0.33	S > R
	ACN	6.34	-	-
Lux Cellulose-4	MeOH	6.73	-	-
	EtOH	6.77	-	-
	IPA	16.07	0.60	S > R
	ACN	9.23	-	-

* All mobile phases contain 0.1% (*v*/*v*) acetic acid.

**Table 2:** Experimental design plan with the obtained results for the selected responses.

	Water Content (*v*/*v* %)	Column Temperature (°C)	Flow Rate (mL/min)	Acetic Acid (*v*/*v* %)	t_2_ (min)	*R*_s_
1	15	40	0.7	0.05	6.14	3.2
2	10	35	0.5	0.1	7.24	2.6
3	10	35	0.5	0.05	7.24	2.6
4	5	30	0.3	0.05	10.07	1.7
5	15	40	0.3	0.05	14.17	3.8
6	10	40	0.5	0.1	6.91	2.5
7	10	35	0.3	0.1	11.92	2.8
8	10	35	0.7	0.1	5.11	2.3
9	5	35	0.5	0.1	5.85	1.6
10	10	35	0.5	0.1	7.24	2.6
11	10	35	0.5	0.1	7.23	2.6
12	10	35	0.5	0.1	7.23	2.6
13	5	30	0.7	0.05	4.30	1.5
14	10	35	0.5	0.1	7.26	2.6
15	10	30	0.5	0.1	7.62	2.6
16	15	35	0.5	0.1	9.37	3.6
17	15	30	0.7	0.15	6.84	3.3
18	10	35	0.5	0.15	7.12	2.6
19	5	40	0.3	0.15	9.38	1.6
20	5	40	0.7	0.15	4.04	1.4
21	15	30	0.3	0.15	16.69	4.2

**Table 3:** Summary of the data obtained during method validation.

Parameter	Result
Linearity	
Range (%)	0.05–3%
Range (μg/mL)	2.5–150
Regression equation	11.932x + 0.2905
Coefficient of determination (r^2^)	0.9998
Sensitivity	
LOD (μg/mL) (S/N = 3)	0.6
LOQ (μg/mL) (S/N = 10)	2.0
Repeatability (six injections on the same day) RSD%	
Level I (5 μg/mL)	1.02
Level II (25 μg/mL)	0.75
Level III (125 μg/mL)	0.63
Intermediate precision (Analysis on three consecutive days, each sample analyzed in triplicate) (RSD%)	
Level I (5 μg/mL)	1.25
Level II (25 μg/mL)	0.89
Level III (125 μg/mL)	1.19
Accuracy (Expressed as mean recovery ± 95% confidence interval (*n* = 3, α = 0.05)) (%).	
Level I (5 μg/mL)	99.06 ± 0.71
Level II (25 μg/mL)	100.42 ± 0.84
Level III (125 μg/mL)	98.97 ± 0.77

**Table 4:** Comparison of the current and previously described methods for the enantioseparation of naproxen.

Column	Mobile Phase	Separation Mode	Enantiomeric Quality Control	*R_s_*	t (min)	Ref.
Lux Amylose-1	MeOH:H_2_O:acetic acid mixture	RP	Yes (0.1% limit)	3.2 (optimized by FCCD)	~7	Current work
Silica gel π-acceptor/π-donor for chiral separations	Hexane:IPA:ACN:acetic acid	NP	Yes (2.5% limit)	~3	~7.5	EurPh
Chiralcel OD	Hexane:IPA:acetic acid	NP	No	1.7	~20	[9]
Chiralpak IC	Hexane:EtOH:TFA	NP	N	3.4	-	[10]
Chiralpak AS-3R	ACN:phosphate buffer	RP	Yes (not complete validation)	2.55	~7	[12]
FLM Chiral NQ(2)-RH	ACN:formic acid in water	RP	Yes	2.38	~14	[18]
